# Muscarinic Acetylcholine Type 1 Receptor Activity Constrains Neurite Outgrowth by Inhibiting Microtubule Polymerization and Mitochondrial Trafficking in Adult Sensory Neurons

**DOI:** 10.3389/fnins.2018.00402

**Published:** 2018-06-26

**Authors:** Mohammad G. Sabbir, Nigel A. Calcutt, Paul Fernyhough

**Affiliations:** ^1^Division of Neurodegenerative Disorders, St. Boniface Hospital Research Centre, Winnipeg, MB, Canada; ^2^Department of Pathology, University of California, San Diego, San Diego, CA, United States; ^3^Department of Pharmacology and Therapeutics, University of Manitoba, Winnipeg, MB, Canada

**Keywords:** muscarinic receptors, mitochondria, antagonist, G proteins, cytoskeleton dynamics, mitochondrial trafficking, neurodegeneration, tubulin

## Abstract

The muscarinic acetylcholine type 1 receptor (M_1_R) is a metabotropic G protein-coupled receptor. Knockout of M_1_R or exposure to selective or specific receptor antagonists elevates neurite outgrowth in adult sensory neurons and is therapeutic in diverse models of peripheral neuropathy. We tested the hypothesis that endogenous M_1_R activation constrained neurite outgrowth via a negative impact on the cytoskeleton and subsequent mitochondrial trafficking. We overexpressed M_1_R in primary cultures of adult rat sensory neurons and cell lines and studied the physiological and molecular consequences related to regulation of cytoskeletal/mitochondrial dynamics and neurite outgrowth. In adult primary neurons, overexpression of M_1_R caused disruption of the tubulin, but not actin, cytoskeleton and significantly reduced neurite outgrowth. Over-expression of a M_1_R-DREADD mutant comparatively increased neurite outgrowth suggesting that acetylcholine released from cultured neurons interacts with M_1_R to suppress neurite outgrowth. M_1_R-dependent constraint on neurite outgrowth was removed by selective (pirenzepine) or specific (muscarinic toxin 7) M_1_R antagonists. M_1_R-dependent disruption of the cytoskeleton also diminished mitochondrial abundance and trafficking in distal neurites, a disorder that was also rescued by pirenzepine or muscarinic toxin 7. M_1_R activation modulated cytoskeletal dynamics through activation of the G protein (Gα13) that inhibited tubulin polymerization and thus reduced neurite outgrowth. Our study provides a novel mechanism of M_1_R control of Gα13 protein-dependent modulation of the tubulin cytoskeleton, mitochondrial trafficking and neurite outgrowth in axons of adult sensory neurons. This novel pathway could be harnessed to treat dying-back neuropathies since anti-muscarinic drugs are currently utilized for other clinical conditions.

## Introduction

Muscarinic acetylcholine receptors constitute a sub-family of G protein-coupled receptors (GPCRs) that act as metabotropic activators of the neurotransmitter acetylcholine (ACh). Five distinct subtypes have been identified (M1-M5), based on their G-protein coupling preferences (Wess, 1996). Downstream pathways activated include phospholipase C, inositol triphosphate (IP_3_), cyclic adenosine monophosphate (cAMP) and altered calcium homeostasis (Eglen, 2005; Wess et al., 2007; Kruse et al., 2014). In addition, these GPCRs modulate the cytoskeleton through trimeric G protein signaling (Kapitein and Hoogenraad, 2015). For example, α and βγ subunits of heterotrimeric G proteins modulate microtubule assembly (Roychowdhury and Rasenick, 2008; Schappi et al., 2014). Activated Gα, acts as a GTPase activating protein (GAP) and increases microtubule disassembly by activating the intrinsic GTPase activity of tubulin (Roychowdhury et al., 1999).

The muscarinic acetylcholine type 1 receptor (M_1_R) is widely expressed in the central nervous system (CNS) (Levey, 1993; Wess et al., 2003; Jiang et al., 2014) and peripheral nervous system (PNS) (Bernardini et al., 1999; Tata et al., 2000b). Membranes isolated from hippocampus and cortex of M_1_R knockout (KO) mice showed a significant decrease in GTPγ-S loading to the Gα-q/11 G protein upon agonist stimulation (Felder et al., 2001). In cortical neuron cultures obtained from M_1_R KO mice, carbachol-stimulated phosphoinositide hydrolysis was reduced by 60% compared with wild type (Bymaster et al., 2003). In addition, phosphorylation of extracellular signal-regulated kinase (ERK) was eliminated in pyramidal neurons of hippocampal slices or cortical cultures derived from M_1_R KO mice (Berkeley et al., 2001; Hamilton and Nathanson, 2001).

In sympathetic neurons, ACh activation of M_1_R mobilizes internal Ca^2+^ stores leading to closure of M-type K^+^ channels (Kv7 subtypes) and enhancement of slow depolarization and discharge (Delmas and Brown, 2005; Brown and Passmore, 2009). In embryonic neurons, ACh modulates neurite outgrowth in a positive or negative manner based upon context (Tata et al., 2000a, 2003; Bernardini et al., 2004; Yang and Kunes, 2004). Furthermore, both adult sensory dorsal root ganglia (DRG) neurons and epidermal keratinocytes synthesize and secrete ACh (Bernardini et al., 1999; Khan et al., 2002; Nguyen et al., 2004; Grando et al., 2006; Schlereth et al., 2006; Corsetti et al., 2012). Adult rat sensory neurons of the DRG express a peripheral form of ChAT (pChAT), exhibit ChAT activity, have low AChE activity and express multiple muscarinic receptors including M_1_R (Bernardini et al., 1999; Tata et al., 2000b; Bellier and Kimura, 2007; Hanada et al., 2013).

We have recently reported that selective or specific antagonists of M_1_R elevated neurite outgrowth and augmented mitochondrial function in adult sensory neurons (Calcutt et al., 2017). These drugs also afforded protection against several different forms of peripheral neuropathy. However, the mechanism of M_1_R antagonist-driven neurite outgrowth and neuroprotection has not been studied in detail. Mitochondrial oxidative phosphorylation is the main mechanism providing ATP to power neuronal activities such as production of presynaptic action potentials, neurotransmitter release, postsynaptic currents and postsynaptic action potentials (Hall et al., 2012). Mitochondria are known to concentrate in regions of active signaling and high metabolic demand (Chen and Chan, 2006; Mironov, 2007; Verburg and Hollenbeck, 2008). This substantial energy demand at the nerve ending or synapse implies that neurons must have a mechanism to maintain microtubules to augment mitochondrial trafficking upon demand (Sheng and Cai, 2012; Schwarz, 2013).

In the present study we manipulated M_1_R expression/function in adult DRG sensory neurons and related cell lines and studied the cellular phenotypes and molecular consequences. Specifically, we tested the hypothesis that the M_1_R regulates the tubulin cytoskeleton, G-protein recruitment (Gα13 sub-type) and mitochondrial trafficking. We identified that excessive cholinergic signaling triggered tubulin destabilization through over-activation of Gα13 proteins. Further, we studied the ability of specific (muscarinic toxin 7: MT7) or selective (pirenzepine) M_1_R antagonists to ameliorate the endogenous and M_1_R overexpression-induced neuronal phenotypes that primarily result in a constraint on neurite outgrowth.

## Materials and Methods

All animal procedures followed guidelines of University of Manitoba Animal Care Committee using Canadian Council of Animal Care rules or of the Institutional Animal Care and Use Committee at UCSD.

### Cell Culture

Dorsal root ganglia from adult male Sprague-Dawley rats were dissected and dissociated using previously described methods (Akude et al., 2011; Roy Chowdhury et al., 2012; Saleh et al., 2013). All animal protocols carefully followed the Canadian Committee on Animal Care (CCAC) guidelines. Neurons were cultured in defined Hams F12 media containing 10 mM D-Glucose (N4888, Sigma, St. Louis, MO, United States) supplemented with modified Bottenstein’s N2 additives without insulin (0.1 mg/ml transferrin, 20 nM progesterone, 100 μM putrescine, 30 nM sodium selenite, 0.1 mg/ml BSA; all additives were from Sigma, St. Louis, MO, United States). In all experiments, the media was also supplemented with 0.146 g/L L-glutamine, a low dose or high dose cocktail of neurotrophic factors (Low dose = 0.1 ng/ml NGF, 1.0 ng/ml GDNF and 1 ng/ml NT-3, High dose = 1 ng/ml NGF, 10 ng/ml GDNF, 10 ng/ml NT3 – all from Promega, Madison, WI, United States), 0.1 nM insulin and 1X antibiotic antimycotic solution (A5955, Sigma). Cultures were treated with 100 nM MT7 (M-200, Alomone Labs, Jerusalem, Israel) or 1 μM pirenzepine (P7412, Sigma).

HEK293 and HTLA cells were cultivated in Dulbecco’s modified Eagle’s medium (DMEM) supplemented with 10% heat inactivated FBS. The β-arrestin null (ARRB1 and ARRB2) and Gα12/13 (GNA12 and GNA13) null HEK293 cells were obtained from the laboratory of Dr. Asuka Inoue, Tohoku University, Japan. HTLA cells were provided by Prof Bryan Roth, University of North Carolina, United States.

### Cloning, Transfection and siRNA Based Gene Silencing

Total mRNA was extracted from adult rat DRGs using Trizol reagent and used for amplifying the full length M_1_R cDNA using the following primer sets: F: 5′-ATGAACACCTCAGTGCCCCCTGC-3′ and R: 5′-TTAGCATTGGCGGGAGGGGGTG-3′. The cDNA was cloned in the pEGFP-C1 vector (Clontech, now Takara Bio United States, Inc., Mountain View, CA, United States) in the XhoI and SacII restriction sites. In addition, the cDNA was also cloned in the pHTN Halo-Tag CMV-neo Vector (Promega, Madison, WI, United States) at the SacII and Not1 restriction sites. The plasmids were transiently transfected in freshly dissociated sensory neurons using the rat neuron nucleofection kit (VPG-1003, Amaxa, Lonza Inc., Allendale, NJ, United States) and Amaxa nucleofector-II apparatus (program 0-003) and cultured in poly L-Ornithine (P8638, Sigma) and laminin coated μ-Plate-24 well (Ibidi United States, Inc., Madison, WI, United States). The human M1-DREADD construct was obtained from Dr. Arthur Christopoulos, Monash University, Australia and sub-cloned in pEGFP-C1 vector (Abdul-Ridha et al., 2013). The rat Gα13(GNA13) was knocked down using a cocktail of three siRNAs targeted to exon 2 (AGTATCTTCCTGCTATAAGAGCC) and exon 4 (CTACATTCCGTCACAGCAAGATA and CATCAAAGACTATTTCCTAGAAT), respectively. The siRNAs were transfected in to primary sensory neurons using Amaxa transfection reagent.

### Quantification of Neurite Outgrowth

The transgene transfected neurons were cultured for 48 h and then cells were fixed in 4% paraformaldehyde for 10 min and immunostained using monoclonal anti-β-tubulin III antibody. The neurons were also stained with Hoechst for nuclear staining. The neurons were imaged in an unbiased manner using a Cellomics Arrayscan-VTI high content screening (HCS) Reader (Thermo Fisher Scientific, Waltham, MA United States) and total neurite outgrowth per neuron was measured by Neuronal Profiling V4.1 software. The automated HCS reader provided a bias-free objective analysis of neurite outgrowth.

### Confocal Microscopic Image Acquisition and Analysis to Determine Mitochondrial Volume and Trafficking

Mitochondrial trafficking in GFP or GFP-M_1_R overexpressing neurons was monitored using LSM510 confocal live cell imaging and involved co-transfection of sensory neurons with GFP/GFP-M_1_R and DsRed2Mito7 plasmids (Addgene plasmid #55838, a gift from Dr. Michael W. Davidson, Florida State University), respectively. The DsRed2Mito7 consists of a mitochondrial targeting sequence from subunit VIII of human cytochrome C oxidase which is placed before the Red fluorescence protein and the resultant fusion protein selectively accumulates inside the mitochondria (Van Kuilenburg et al., 1988). The transfected cells were live imaged at 10 s interval for 80 time frames (∼13 min). The time lapse images were used to generate kymographs using the ImageJ make kymograph plugin (Schneider et al., 2012). In each kymograph, the *x*-axis represents the position along the length of the axon and the *y*-axis represents time. Vertical lines indicate stationary mitochondria with no displacement during the time elapsed and diagonal lines represent moving mitochondria and their direction. Their velocity is reflected in the slope of the line. In addition we used Fiji (Schindelin et al., 2012) based MTrackJ to determine mitochondrial velocity (μm/sec) in the neurites (Meijering et al., 2012). The volume of mitochondria in the neurites was calculated by using Image J analyze particles plugin and expressed in μm per neurite length (Schindelin et al., 2012).

### Western Blotting and Immune-Detection

Relative quantification of proteins was done by SDS-PAGE separation of total proteins followed by transfer to nitrocellulose membrane and immunoblotting based detection using HRP-conjugated secondary antibodies. The immunoblots were imaged in Bio-Rad Chemidoc system (Bio-Rad Laboratories Ltd., Mississauga, ON, Canada). **Table 1** summarizes all the primary antibodies used in this study. The cell lysates were prepared in 1X RIPA lysis and extraction buffer (Cat No: 89900, Thermo Fisher Scientific) supplemented with 1X Halt protease and phosphatase inhibitor cocktail (Cat No: 78441, Thermo Fisher Scientific).

**Table 1:** List of antibodies.

Name of the antibody; clone number	Catalog number	Host species and type	Vendor
Anti-porin (B-6)	Sc-390996	Mouse monoclonal	SCBT
Anti-β-actin (C-4)	Sc-47728	Mouse monoclonal	SCBT
Anti GAPDH (0411)	Sc-25778	Mouse monoclonal	SCBT
Anti-α-tubulin (TU-02)	Sc-8035 (TU-02)	Mouse monoclonal	SCBT
Anti-GFP	Sc-9996	Mouse monoclonal	SCBT
Anti-M_1_R	Sc-365966	Mouse monoclonal	SCBT
Anti-Gγ2/3/4/7	Sc-166419	Mouse monoclonal	SCBT
Anti-Gα12/13	Sc-28588	Mouse monoclonal	SCBT
Anti-β-tubulin III	T8578	Mouse monoclonal	Sigma

*SCBT, Santa Cruz Biotechnology.*

### Polymerized Tubulin Quantification

The polymerized tubulin in M_1_R overexpressed cells (40–50% transfection efficiency) was quantified by methods described previously (Butcher et al., 2016). Briefly, the soluble fraction of tubulin was first removed by lysing the cells in a microtubule stabilizing buffer (MSB) containing 50% glycerol, 5 mM MgCl_2_, 0.1 mM EGTA, 0.3 mM guanosine triphosphate (grade II-S, Sigma Chemical Co.), and 10 mM sodium phosphate, pH 6.8 (Beertsen et al., 1982). Cells were harvested with a rubber scraper in MSB, homogenized, and centrifuged at 26,000 × *g* in a Sorvall RC2-B centrifuge (17,000 rpm in rotor SS-34 in 1.0 ml tubes; Dupont Instruments-Sorvall Biomedical Div., Dupont Co., Newtown, CT, United States) at 20°C for 30 min. Supernatants containing the soluble tubulin fraction were removed and the pellet containing polymerized tubulin and other cytoskeletal protein was assessed by immunoblotting using anti-actin and anti-β tubulin III antibodies.

### Halo-M_1_R Pull Down and Blue-Native Polyacrylamide Gel Electrophoresis (BN-PAGE)

SH-SY5Y human neuroblastoma cells (provided by Dr. Jun-Feng Wang, University of Manitoba) were grown in dulbecco’s modified eagle medium: nutrient mixture F-12 (DMEM/F12, Thermo Fisher) supplemented with 10% fetal bovine serum (FBS, Thermo Fisher). Halo-M_1_R plasmid was transiently expressed in SH-SY5Y cells that were treated with 100 nM MT7 or 1 μM pirenzepine for 1 h. Halo-M_1_R was then pulled down using Halo-link resin by overnight incubation at 4°C (Promega Corporation, Madison, WI, United States) as per manufacturer’s instruction and the pull-down product was cleaved using TEV-protease (Promega) overnight on a constant rotating shaking platform. The cleaved fraction was resolved in SDS-PAGE and immunoblotted using anti-M_1_R, anti Gγ2/3/4/7 and anti-Gα12/13 antibodies. The BN-PAGE was performed as described previously (Sabbir et al., 2016). Polymeric tubulins in Gα12/13 null and native HEK293 cells were separated by BN-PAGE using microtubule stabilizing native cell lysis buffer containing 20 mM Bis-tris (pH7.0), 500 mM ε-aminocaproic acid, 20 mM NaCl, 10% Glycerol, 5 mM MgCl_2_, and 0.3 mM GTP (Beertsen et al., 1982).

### Isoelectric Focusing

Fifty microgram of total cellular protein was precipitated by acetone and dissolved in rehydration buffer containing 8 M Urea, 2% CHAPS, 50 mM dithiothreitol (DTT) and 0.2% Bio-Lyte ampholytes pH3-10. The dissolved proteins were incubated in Zoom IPG-strip 3-10 non-linear (NL) (Thermo Fisher) for 1 h and then focused at 175 volt (V) for 15 min, 175–2000 V ramp for 45 min and 2000 V for 30 min. After the run, the strips were alkylated and resolved on 2D SDS-PAGE and immunoblotted using antibodies previously described.

### Statistical Analysis

Statistical analysis was performed using Prism version 7.00 (GraphPad Software). The mean of two or more groups were compared using one-way ANOVA followed by multiple comparison tests (Siegel, 1956; Dunn, 1964). The mean of multiple experimental groups were compared with the control group by Dunnett’s *post hoc* multiple comparison test, whereas, the mean difference between two experimental groups were compared by Sidak’s *post hoc* multiple comparison test (Dunn, 1964). Comparisons between two groups were performed using Student’s *t*-test (unpaired). Differences were considered significant at *P* < 0.05. Neurite outgrowth, neurite width and mitochondrial quantification data were plotted as box and whisker plot where the end of the box represents the upper and lower quartiles and the median is marked by a horizontal line inside the box. The whiskers represent the highest and lowest values excluding the outliers. In some figures the individual data points of outliers are also indicated.

## Results

### MT7 and Pirenzepine Significantly Augmented Neurite Outgrowth in Cultured Primary Sensory Neurons

**FIGURE 1:**
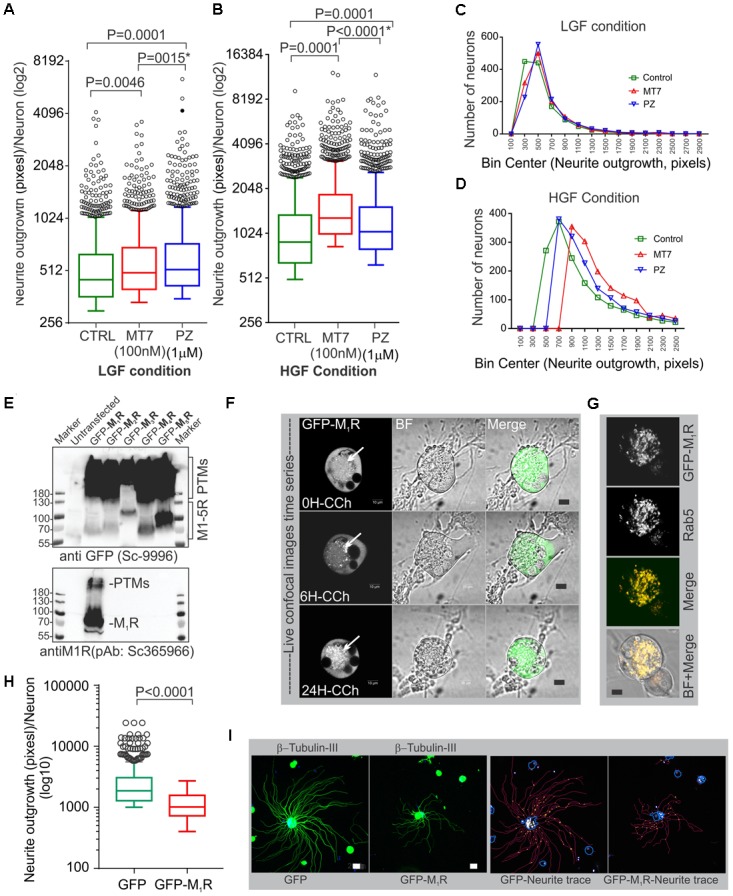
M_1_R antagonists, MT7 and pirenzepine, augment neurite outgrowth in primary sensory neurons and M_1_R overexpression inhibits neurite outgrowth. **(A,B)** Whiskers box (Tukey) showing total neurite outgrowth per neuron. Neurons were grown for 48 h in defined media containing LGF **(A)** or HGF **(B)** condition and treated with 100 nM MT7 or 1 μM pirenzepine (PZ), respectively. *N* = 1249 (LGF) and 1517 (HGF), respectively. *P*-values were calculated by one-way ANOVA followed by *post hoc* multiple comparison tests. Dunnett’s multiple comparisons test was used to compare the MT7 and PZ treatment groups with the control group and Sidak’s multiple comparisons test was used to compare between the MT7 and PZ treatment groups; ^∗^ indicates the *p*-value obtained by Sidak’s multiple comparisons test. **(C,D)** Binning of the entire data set presented in **(A,B)**. **(E)** Immunoblot showing GFP-tagged muscarinic receptors (M_1_R to M_5_R) and GFP expression in transfected adult rat DRG neurons. pEGFP-C1-(M_1_R-M_5_R) plasmids were transfected in to DRG neurons and the lysate was resolved in SDS page and subsequently immunoblotted with anti-M_1_R (bottom panel) and anti-GFP (top panel) antibodies. **(F)** Time lapse confocal images showing increasing internalization (white arrows) of the GFP-M_1_R following treatment with carbachol (10 μM). Scale bar: 10 μm. **(G)** Immunofluorescence images showing colocalization of 24h CCh treated GFP-M1R with endosomal marker Rab5. Scale bar: 10 μm. **(H)** Whiskers box (Tukey) showing total neurite outgrowth per neuron, *N* = 634 (GFP), and *N* = 553 (GFP-M_1_R), neurons, respectively. *P*-value was calculated by *t*-test (unpaired). **(I)** Immunofluorescence images showing β-tubulin III staining and corresponding neurite trace (red lines) images in GFP and GFP-M_1_R overexpressed neurons. The total neurite outgrowth measurement was performed in Cellomics ArrayScan HCS Reader using neuronal profiling software. Scale bar: 10 μm.

Pirenzepine is a selective M_1_R antagonist whereas MT7 is the only specific antagonist of this receptor (Birdsall et al., 1983; Max et al., 1993a). Our previous study demonstrated that both antagonists enhanced neurite outgrowth from adult sensory neurons (Calcutt et al., 2017). In order to confirm that MT7 and pirenzepine had growth promoting effects under the current conditions, we measured total neurite outgrowth from primary sensory neurons derived from adult rats and cultured in defined media containing a cocktail comprising of low or high concentrations of GDNF, NGF, and NT3 growth factors (LGF and HGF, respectively) and either 100 nM MT7 or 1 μM pirenzepine (**Figures 1A–D**). The growth factor concentrations in the LGF cocktail reflects concentrations of growth factors that are sub-saturating and induce small but significant increases in neurite outgrowth (Calcutt et al., 2017). The HGF cocktail contained 10-fold higher concentrations to allow us to determine whether the neuritogenic effects of MT7 and pirenzepine were also effective when growth factors were present in excess and, also to see if increased tyrosine kinase signaling masked the effect of antagonist-M_1_R signaling mediated growth. In the absence of muscarinic antagonists, the HGF promoted significantly (*p* < 0.0001) more neurite outgrowth than LGF, as assessed using unbiased automated high content imaging combined with data analysis (**Figures 1A,B**). Both MT7 and pirenzepine significantly increased neurite outgrowth in LGF and HGF conditions within 48 h of treatment (**Figures 1A,B**). In addition, binning of the entire data set of 1249 (LGF) and 1517 (HGF) neurons for their total neurite length revealed that the neuritogenic effect of pirenzepine and MT7 evenly affected neurite outgrowth of the majority of the population of neurons (**Figures 1C,D**). Further, pirenzepine treatment elicited significantly higher neurite outgrowth under the LGF conditions when compared to MT7, whereas under the HGF condition, the primacy was reversed (**Figures 1A,B**). The exact reason for the difference between the drugs in terms of synergistic effect on growth at HGF condition is not known. The difference in the chemical nature of these drugs may be responsible. MT7 is a cell impermeable 7.4 kDa protein (Krajewski et al., 2001) which binds allosterically to M_1_R (Max et al., 1993b; Karlsson et al., 2000) whereas pirenzepine is a cell-permeable orthosteric antagonist molecule (Caulfield and Birdsall, 1998). We used LGF conditions in subsequent experiments.

### M_1_R Overexpression Induced Significant Reduction in Neurite Outgrowth

**FIGURE 2:**
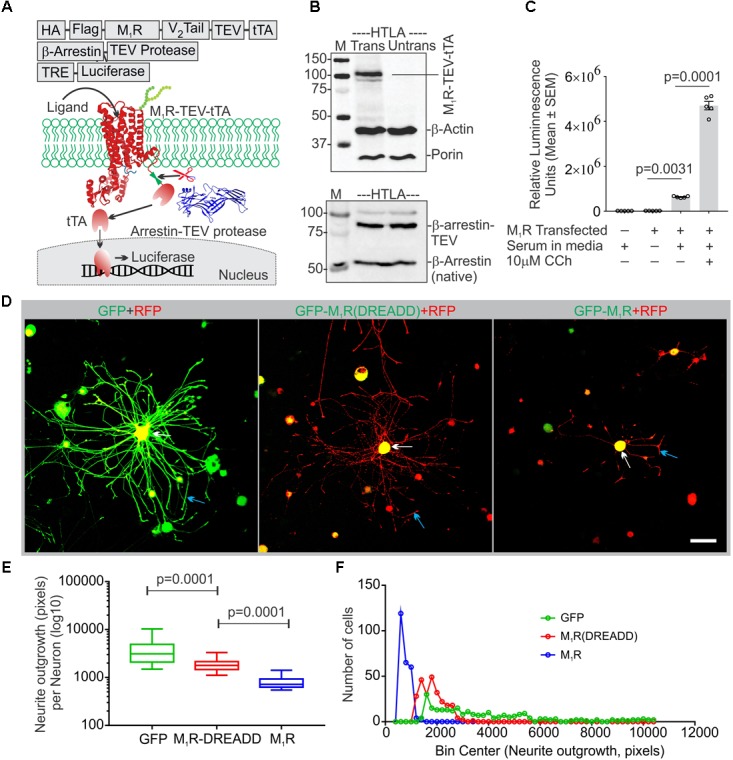
Constitutive basal activity of M_1_R and the effect of DREADD mutant on neurite outgrowth. **(A)** Graphical representation of the arrestin recruitment assay (TANGO) strategy, TRE-Tet response element. HA-cleavable signal sequence to promote membrane localization, FLAG-epitope tag, TEV- Tobacco Etch Virus cleavage site, V_2_ tail: C-terminus of the V_2_ vasopressin receptor (V_2_ tail) to promote arrestin recruitment and tTA- tetracyclin transactivator. **(B)** Immunoblot showing expression of M_1_R-TEV-tTA and β-arrestin-TEV protease transgene. **(C)** Scatter plot showing the RLU (Relative Luminescence Units) for the drug treated HTLA cells. *N* = 5 independent experiments. **(D)** Co-expression of GFP/GFP-M_1_R/GFP-M_1_R(DREADD) with mito7-RFP in DRG neurons following 48 h of growth. Neurite outgrowth suppression was comparatively less in M_1_R-DREADD mutant over-expressing neurons. The white and blue arrows indicate that localization of GFP-M_1_R was mainly restricted in the perikaryon where as GFP was localized both in perikaryon and neurites. LSM510 confocal images acquisition parameters were same for all images. Scale bar: 100 μm. **(E)** Whiskers box (Tukey) showing total neurite outgrowth per neuron. *N* = 250. *p*-value by one-way ANOVA followed by Dunnett’s multiple comparisons test. **(F)** Binning of the entire data set presented in **(E)**.

The muscarinic receptor subtypes (M1–M5) show considerable heterogeneity of expression in sensory neurons(Bernardini et al., 1999; Chiu et al., 2014). In order to understand the biological function of M_1_R in sensory neurons, we overexpressed GFP-tagged M_1_R in sensory neurons and measured the impact on neurite outgrowth. M_1_R overexpression significantly reduced neurite outgrowth when compared to GFP-expressing neurons (**Figures 1H,I**). The GFP-M_1_R transgene-induced protein production was verified in the transfected DRG neurons by immunoblotting of the expressed recombinant proteins (GFP-M1-5R) using both anti-M_1_R and anti-GFP antibodies (**Figure 1E**). The biological functionality of the GFP-M_1_R recombinant protein was verified by treating GFP-M_1_R overexpressing DRG neurons with the broad spectrum muscarinic agonist carbachol (10 μM) followed by live confocal imaging to monitor internalization of recombinant protein. Treatment with carbachol increased presence of the recombinant protein as internalized spots that were positive for the early endosomal marker Rab5 (Zerial and McBride, 2001) (**Figures 1F,G**). This indicates that GFP-tagged recombinant M_1_R elicited agonist induced internalization response similar to that of naïve proteins. In addition, to assess functionality of the recombinant M_1_R in overexpressed cells, we performed a β-arrestin recruitment assay [TANGO assay: (Kroeze et al., 2015), **Figure 2**]. In the TANGO assay, upon activation, β-arrestin is recruited to the C-terminus of the M_1_R-TEV-tTA fusion protein at the TEV protease site and cleaves to release the tTA transcription factor, which after transport to the nucleus activates transcription of luciferase reporter gene (Kroeze et al., 2015) (**Figure 2A**). We verified expression of the M_1_R-TEV-tTA and β-arrestin transgenes by immunoblotting (**Figure 2B**). We found significant recruitment of β-arrestin due to basal M_1_R activation in presence of serum in the culture media in the HTLA cells (**Figure 2C**). This basal activation of M_1_R occurred in the absence of exogenously supplied agonist. However, fetal bovine serum (FBS) used in the culture media is reported to contain acetylcholine (ACh) (Lau et al., 2013) and this may have constitutively acted upon the M_1_R. Treatment with the muscarinic agonist, carbachol, significantly increased activation of the M_1_R by up to ∼7.4-fold (**Figure 2C**).

### Endogenous ACh Binds M_1_R to Constrain Neurite Outgrowth

Adult sensory neurons secrete ACh into the extracellular media when grown in culture (Calcutt et al., 2017). The Tango Assay revealed that ACh present in the serum supplied to the culture media may also act upon M_1_R and constitutively activate the receptor. We therefore hypothesized that overexpression of M_1_R in sensory neurons may lead to increased constitutive activation of the receptor by recruitment of trimeric G-proteins, including the Gα12/13 subtype that promotes tubulin destabilization (Roychowdhury et al., 1999). In order to test this hypothesis we transfected DRG neurons with an M_1_R-DREADD (designer receptors exclusively activated by designer drugs) mutant to eliminate the putative basal activity in M_1_R-DREADD overexpressing neurons (**Figures 2D,E**). The M_1_R-DREADD mutant contained two mutations in the conserved orthosteric site residues (Y106C and A196G in the M_1_R) that minimize responsiveness to ACh (Abdul-Ridha et al., 2013). Overexpression of GFP-M_1_R and M_1_R-DREADD caused low levels of neurite outgrowth (**Figures 2D–F**). However, neurite outgrowth in M_1_R-DREADD neurons was significantly higher than GFP-M_1_R alone over 72 h of culture (**Figures 2D–F**). This indicates that endogenous ACh may interact with M_1_R to limit neurite outgrowth.

### Impact of M_1_R Overexpression on Mitochondrial Abundance

**FIGURE 3:**
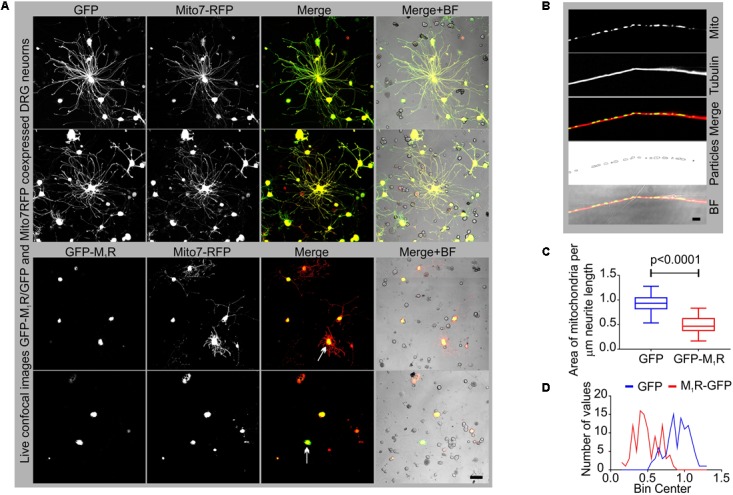
GFP-M_1_R over-expression in sensory neurons caused reduced mitochondrial abundance. **(A)** Representative images showing the signal of co-expression of GFP/GFP-M_1_R and mito7-RFP in DRG neurons. BF, bright field. **(B)** Immunofluorescence images showing mito7-RFP and β-tubulin III stained neurites. The 4th panel image showing tracing of the mitochondrial volume used for determining the amount of mitochondria per unit length of the neurites in GFP/GFP-M_1_R overexpressed DRG neurons. The tracing image was created using ImageJ particle counter plugin. Scale bar: 5 μm. **(C)** Whiskers box (min–max) showing amount of mitochondria in GFP/GFP-M_1_R transfected neurons. *N* = 101 from three independent experiments, *p*-value by *t*-test (unpaired). **(D)** Binning of the entire data set presented in **(C)**.

In an attempt to understand the mechanism of the growth inhibitory effect of M_1_R overexpression, we examined the abundance of mitochondria in the neurites by co-expressing DsRed2Mito7 plasmid (**Figure 3A**). The DsRed2Mito7 plasmid expresses DsRed protein tagged with mitochondrial targeting sequence from subunit VIII of human cytochrome C oxidase and therefore localizes specifically to mitochondria. We measured the volume of mitochondria present per unit length (μm) of neurites in the M_1_R and GFP overexpressing DRG neurons (**Figure 3B**). M_1_R overexpression significantly reduced the abundance of mitochondria in the neurites after 48 h of culture (**Figures 3C,D**). The mitochondria in both M_1_R and GFP over-expressing neurons accumulated mitotracker CMXRos dye, indicating that functionality of the mitochondria was not impaired in M_1_R overexpressed neurons despite reduced abundance (data not shown).

### Reduced Mitochondrial Abundance Was Associated With Impaired Cytoskeletal Structure

**FIGURE 4:**
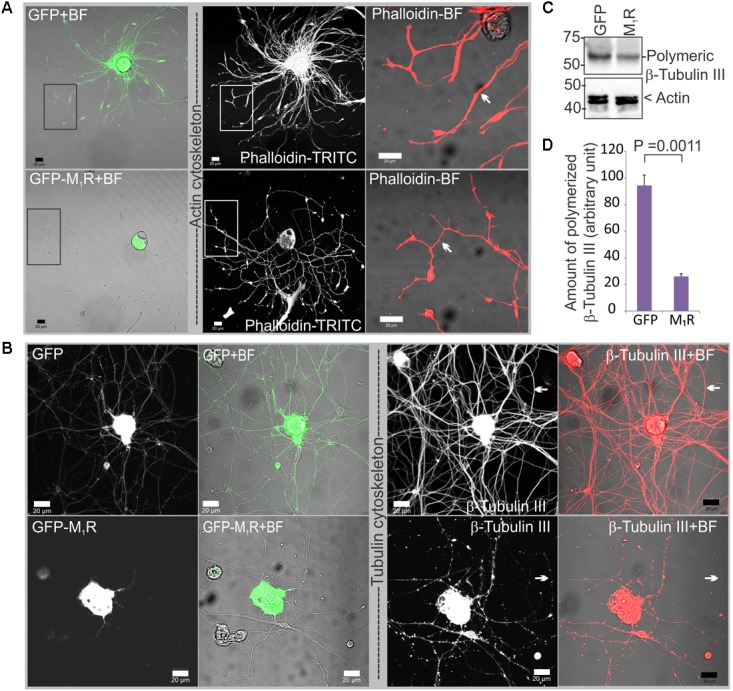
Dorsal root ganglia (DRG) neurons over-expressing M_1_R exhibited cytoskeletal defects. **(A)** Actin cytoskeleton in GFP-M_1_R and GFP over-expressing DRG neurons. White arrow indicates narrower neurites in GFP-M_1_R expressing neurons. **(B)** β-tubulin III associated cytoskeleton in GFP-M_1_R and GFP over-expressing neurons. Black rectangular area is shown in a magnified view in the right panel; white arrow indicates discontinuous/continuous tubulin cytoskeleton. BF, bright field. Scale bar: 20 μm. **(C)** Immunoblot and **(D)** bar graph showing relative amount of polymerized tubulin in the M_1_R-GFP and GFP over-expressed neurons. The data represent the mean ± SEM of three independent experiments. *P* = 0.0011 calculated by unpaired *t*-test.

To determine whether reduced mitochondrial abundance was related to a defect in the actin or tubulin cytoskeletons, we immunostained DRG neurons that overexpressed GFP/GFP-M_1_R with phalloidin and anti-β-tubulin III antibodies. In the presence of M_1_R over-expression, immunofluorescent imaging of phalloidin revealed the presence of abundant and continuous actin filaments in the neurites, although the neurite tips were notably thinner compared to those of GFP expressing neurites (**Figure 4A**). In contrast, the β-tubulin III associated cytoskeleton appeared less abundant and discontinuous (**Figure 4B**). Fragmentation of the tubulin cytoskeleton was confirmed using a polymerized tubulin quantification assay in which there was significantly less polymerized tubulin in the DRG neurons overexpressing M_1_R compared to those that overexpressed only GFP (**Figures 4C,D**).

### Discontinuous Tubulin Cytoskeleton Was Associated With Reduced Mitochondrial Trafficking in Sensory Neurons Overexpressing M_1_R

**FIGURE 5:**
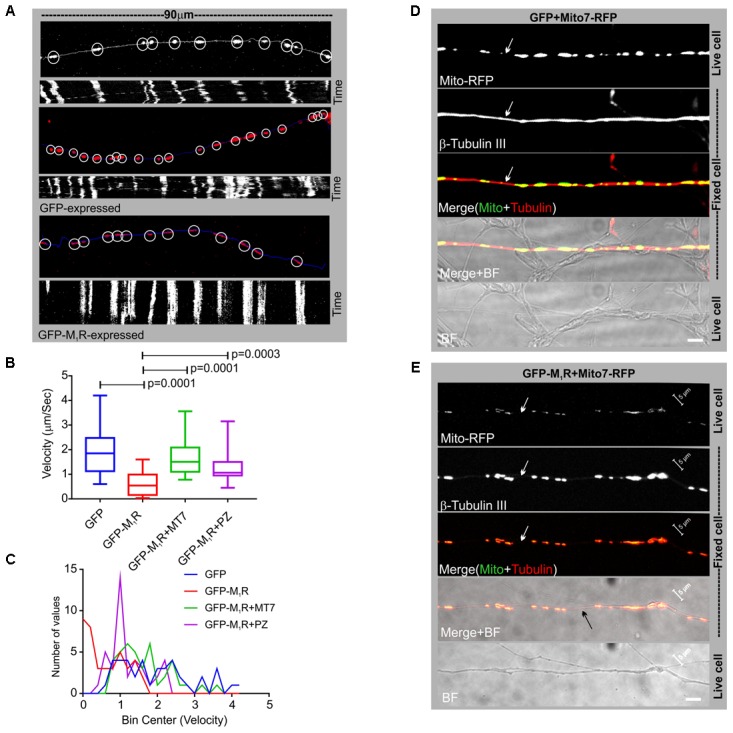
Altered mitochondrial trafficking in M_1_R overexpressing neurons was rescued by antagonist treatment. **(A)** Representative images (top panels) of the first frame of a series of live cell time-lapse images showing the expression of DsRed2Mito7 in the mitochondria of GFP/M_1_R–GFP expressing neurons. The circles represent mitochondria identified by MTrackJ plugin, which then tracked migration through time in a series of time lapse images and calculated velocity of specific mitochondria. White/blue line represents neurite trace. The bottom panel represents Kymographs generated from live cell time-lapse images. The Kymograph was generated using ImageJ Kymograph plugin. The *X*-axis represents the physical location of mitochondria on the neurite, and the *Y*-axis represents the location of mitochondria in time. Streak of particles traversing the kymograph from left to right in angular lines indicates retrograde/anterograde mitochondrial motion. **(B)** Whisker plot showing mitochondrial velocity. DRG neurons were cultured in LGF media supplemented with 100 nM MT7 or 1 μM pirenzepine for 48 h following transfection. *N* = 40 from three independent experiments, *p*-value by one-way ANOVA followed by Dunnett’s multiple comparisons test. **(C)** Binning of the entire data set presented in **(B)**. **(D,E)** Immunofluorescence images showing mito7-RFP and β-tubulin-III staining in the GFP/GFP-M_1_R expressing neurites. White arrows indicate continuous/discontinuous tubulin cytoskeleton in GFP/GFP-M_1_R expressing neurites, respectively. Scale bar: 5 μm.

In order to determine whether the discontinuous β-tubulin cytoskeleton was associated with altered mitochondrial trafficking in growing neurites, we measured velocity of mitochondrial movement in the neurites of DRG neurons that co-expressed GFP/GFP-M_1_R and DsRed2Mito7. Time-lapse images of the neurites at 10 s intervals were used to generate kymographs of mitochondrial trafficking (**Figure 5A**). The mean velocity of mitochondria was calculated as 1.9 μm/sec in GFP expressing neurons whereas in GFP-M_1_R expressing neurons it was significantly lower at 0.62 μm/sec (**Figures 5B,C**). In addition, immunostaining of neurites with a similar appearance to those used in the mitochondrial velocity measurement revealed a continuous β-tubulin-III cytoskeleton in GFP over-expressed neurons and a discontinuous β-tubulin-III cytoskeleton in M_1_R over-expressed neurons (**Figures 5D,E**).

### Muscarinic Antagonists MT7 and Pirenzepine Rescued the Cytoskeletal Defect, Aberrant Mitochondrial Distribution and Trafficking in Neurites

**FIGURE 6:**
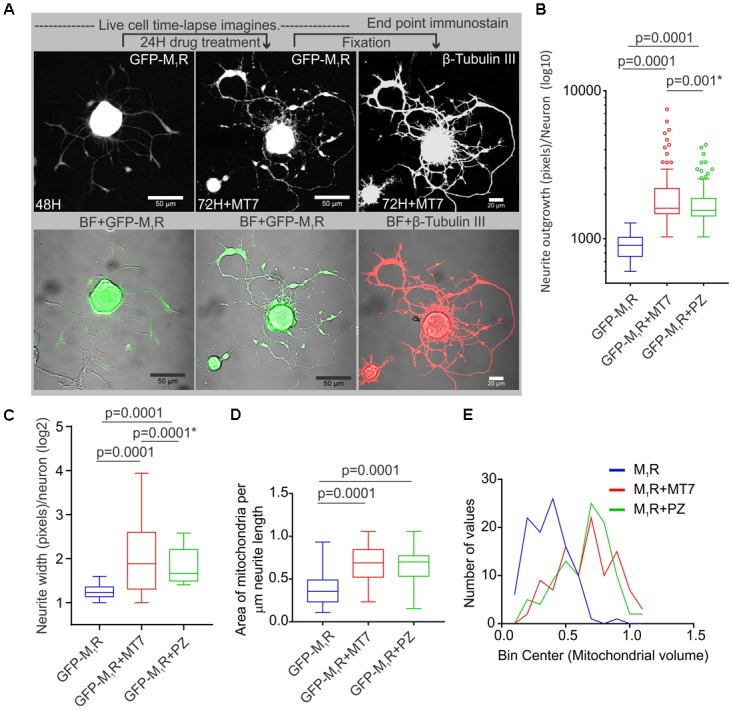
Restoration of cytoskeleton, mitochondrial abundance and neurite outgrowth by M_1_R antagonists MT7 and pirenzepine treatment. **(A)** Time-lapse live confocal images of GFP-M_1_R over-expression in sensory neurons showing MT7 induced re-localization of M_1_R from perikaryon to neurites. The overexpressed neurons were grown in defined media for 48 h and imaged (left panel). Neurons were then treated with 100 nM MT7 for 24 h and imaged (middle panel). Right panel: The same neuron depicted left was fixed and stained for β-tubulin III to show continuity of cytoskeleton. Scale bars: 50 μm. **(B,C)** Whiskers box (Tukey) showing total neurite outgrowth per neuron **(B)** and average neurite width per neuron **(C)**. *p*-value calculated by one-way ANOVA, *N* = 224, 230 and 198 cells, respectively, for **(A)** and *N* = 1250, 1268, 1280, and 1306 cells, respectively, for **(B)**. Asterisks indicate *p*-value calculated by unpaired *t*-test. **(D)** Whisker box plot showing amount of mitochondria in the M_1_R expressing neurons treated with 100 nM MT7 or 1 μM pirenzepine. *N* = 101, *p*-values were calculated by one-way ANOVA followed by Dunnett’s multiple comparisons tests. **(E)** Binning of the entire data set presented in **(D)**.

We investigated whether pirenzepine or MT7 could overcome M_1_R overexpression-induced cytoskeletal defects. M_1_R over-expressing neurons were maintained for 48 h and then treated for 24 h with 100 nM MT7 or 1 μM pirenzepine and total neurite outgrowth and mitochondrial velocity (**Figure 5**) and abundance (**Figure 6**) quantified. MT7 and pirenzepine treatment significantly rescued the deficits in the mitochondrial velocity in M_1_R overexpressed neurons, with mean mitochondrial velocities of 0.78/1.2 μm/s in MT7/pirenzepine treated neurons being significantly higher than untreated neurons (**Figures 5B,C**). Both MT7 and pirenzepine also caused considerable re-localization of M_1_R from the perikarya to the neurites, as revealed by time lapse confocal live cell images over a period of 72 h. This may indicate increased vesicular transport of internalized M_1_R (**Figure 6A**). Within the same neuron (shown in **Figure 6A**), following 24 h of drug treatment and upon fixation and immunostaining for β-tubulin III, there was continuity in the β-tubulin III associated cytoskeleton (right panel). MT7 or pirenzepine significantly increased total neurite outgrowth, reversed the reduced neurite caliber and increased mitochondrial abundance in DRG neurons that overexpressed GFP-M_1_R (**Figures 6B–E**).

### Knockdown of Gα13 in Sensory Neurons Reversed the M_1_R Overexpression-Induced Tubulin Cytoskeleton Defect

**FIGURE 7:**
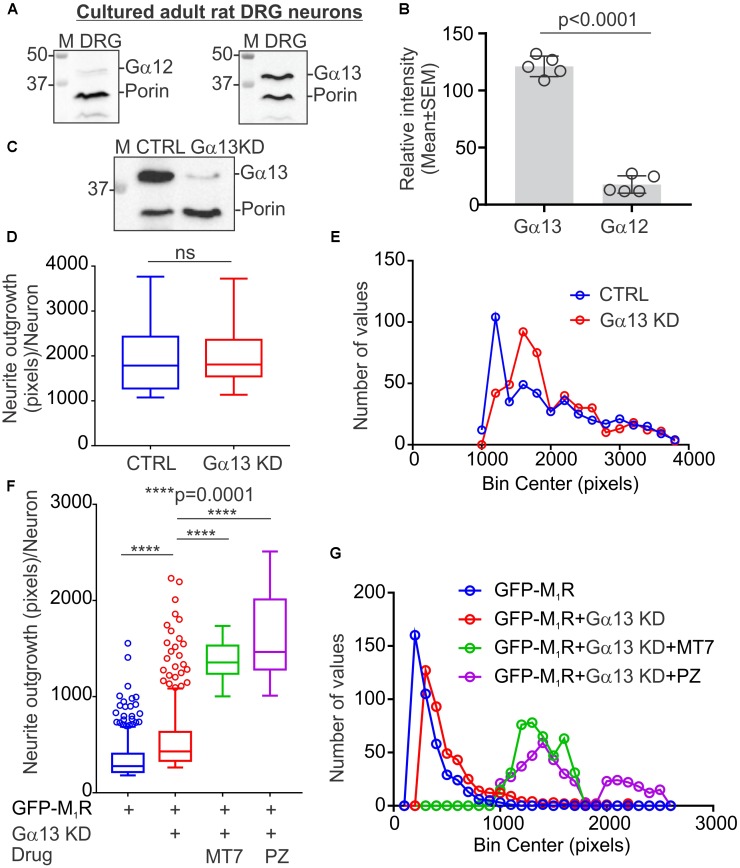
Knockdown of Gα13 reversed M_1_R overexpression-induced inhibition of neurite outgrowth. **(A)** Immunoblots showing relative expression of Gα12 and Gα13 proteins in cultured DRG neurons. **(B)** Scatter plot showing relative amount of Gα12 and Gα13 proteins in cultured sensory neurons. *N* = 5 independent experiments. *p*-value was calculated by unpaired *t*-test. **(C)** Immunoblots showing siRNA (cocktail of 3 siRNAs targeted to rat Gα) based knockdown of Gα13 protein in cultured adult rat DRG neurons. **(D,F)** Whisker box (Tukey) showing total neurite outgrowth per neuron. DRG neurons were transfected with a pEFGP-C1-M_1_R plasmid and siRNA using Amaxa nucleofection reagent and allowed to grow for 48 h. Scrambled siRNAs were used for control. In drug treatment groups, neurons were cultured in media supplemented with 100 nM MT7 or 1 μM pirenzepine following transfection. The neurons were fixed after 48 h of culture, stained with β-tubulin III and imaged using Cellomics ArrayScan HCS Reader. *p*-value by unpaired *t*-test or one-way ANOVA test followed by Dunnett’s multiple comparisons tests. *N* = 432/452 (in **D**) and 406/694 (in **F**). **(E,G)** Binning of the entire data set presented in **(D,F)**.

The M_1_R-DREADD mutant study raised the possibility that, in normal DRG neurons, basal M_1_R activity resulting from binding of endogenous ACh release may destabilize the tubulin cytoskeleton through increased active G proteins. Gα12/13 proteins are known for their effect on cytoskeleton remodeling and the M_1_R receptor activates Gα12/13 type G proteins leading to mobilization of the small GTP-binding protein Rho through activation of Rho-GEF (RhoGTPase nucleotide exchange factor) (Haga, 2013). We, therefore, measured the relative expression of Gα12 and Gα13 proteins in sensory neurons (**Figure 7A**). Immunoblots revealed that DRG neurons express significantly more Gα13 compared to Gα12 (**Figures 7A,B**). The relative levels of Gα12 and Gα13 expression were comparable to those of the human carcinoma cell line HEK293 (**Supplementary Figure S1A**). We used siRNA to knockdown Gα13 in DRG neurons and used a CRISPR/Cas9 based Gα12/13 null HEK293 cell line to study the effect of cholinergic signaling through Gα13 on the tubulin cytoskeleton (**Figure 7C** and **Supplementary Figure S1A**). The HEK293 cell line has been reported to exhibit a neuronal lineage phenotype and express neuronal proteins (Stepanenko and Dmitrenko, 2015). Therefore, we considered it suitable for this study. The siRNA based knockdown of Gα13 in DRG neurons had no significant effect on neurite outgrowth (**Figures 7D,E**). Interestingly, knockdown of Gα13 in M_1_R overexpressed sensory neurons significantly reversed the suppressed neurite outgrowth (**Figures 7F,G**). In addition, treatment of the M_1_R overexpressed and Gα13 knockdown neurons with 100 nM MT7 or 1 μM pirenzepine exhibited significantly increased neurite outgrowth (**Figures 7F,G**).

### CRISPR/Cas9 Based Gα12/13 Null HEK293 Cells Showed Abundant Tubulin Cytoskeleton but Diminished Actin Stress Fibers

RNA sequencing data in the human protein atlas (HPA) indicates that HEK293 cells express very high levels of α-tubulin compared to β-tubulin III (Uhlen et al., 2005). The transcripts per million bases (TPM) value for α-tubulin (TUBA1B isoform) and β-tubulin III has been recorded as 1923.6 and 13.7, respectively^1^. β-tubulin III was undetectable by immunoblotting in HEK293 cells (data not shown). We, therefore, studied α-tubulin dynamics in CRSIPR/Cas9 based Gα12/13 knockout HEK293 cells. We performed immunofluorescent labeling using phalloidin and anti-α-tubulin specific antibodies to visualize the actin and tubulin based cytoskeletal structures in Gα12/13 knockout HEK293 cells (**Supplementary Figure S1E**). Phalloidin staining revealed that actin stress fibers were diminished in Gα12/13 null cells, with appearance of abundant distinct punctate actin rich focal adhesion points (**Supplementary Figure S1E**). Further, the tubulin cytoskeleton appeared more robust and organized in Gα12/13 null cells (**Supplementary Figures S1E**, **S2**). The BN-PAGE based microtubule fractionation assay and polymerized microtubule quantitative assay showed Gα12/13 null cells exhibited significantly more polymerized tubulin than wild type HEK293 cells (**Supplementary Figures S1B–D**). Overexpression of M_1_R in Gα12/13 null cells and treatment with muscarinic agonist carbachol did not alter the tubulin networks as compared to wild type cells (**Supplementary Figures S2**, **S3**).

### MT7 and Pirenzepine Modulate G Protein Interaction With the M_1_R

**FIGURE 8:**
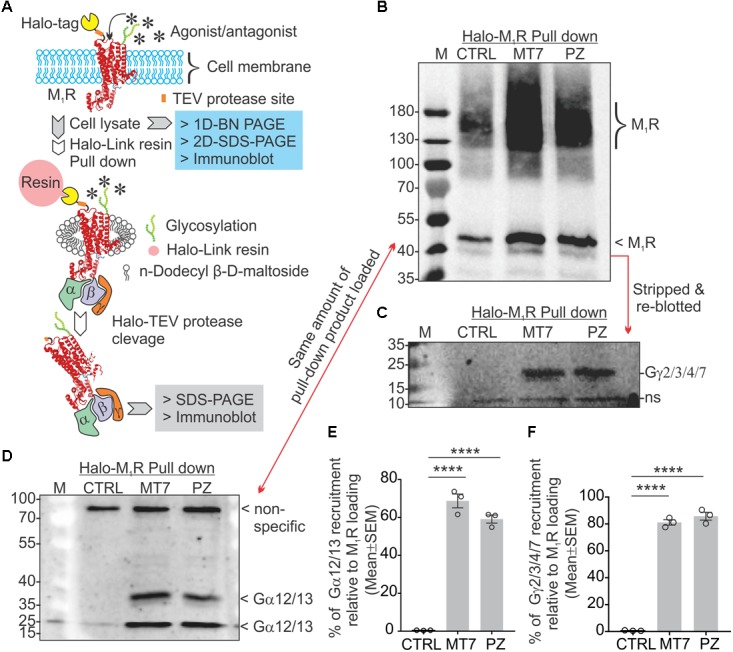
MT7 and pirenzepine elevated sequestration of trimeric G proteins associated with M_1_R in SH-SY5Y cells. **(A)** Diagrammatic representation of the experimental strategy. **(B–D)** Halo-tagged M_1_R was expressed transiently in sensory neurons and then treated with 100 nM MT7 or 1 μM PZ for 1 h. Cells were then lysed and Halo-M_1_R was pulled down using halo-linked resin. The halo tag was cleaved by TEV protease and the cleaved M_1_R associated multiprotein complex (MPC) was resolved in denaturing SDS-PAGE and immunoblotted using **(B)** anti-M_1_R, **(C)** anti-Gγ2/3/4/7, and **(D)** anti-Gα12/13 antibodies. **(E,F)** Scatter plot showing the relative amount of G proteins (Gα and Gγ, respectively) associated with M_1_R following drug treatment. The data represent mean ± SEM of three independent experiments. *p*-values (^∗∗∗∗^<0.0001) were calculated by one-way ANOVA with Dunnett’s *post hoc* multiple comparison test.

**FIGURE 9:**
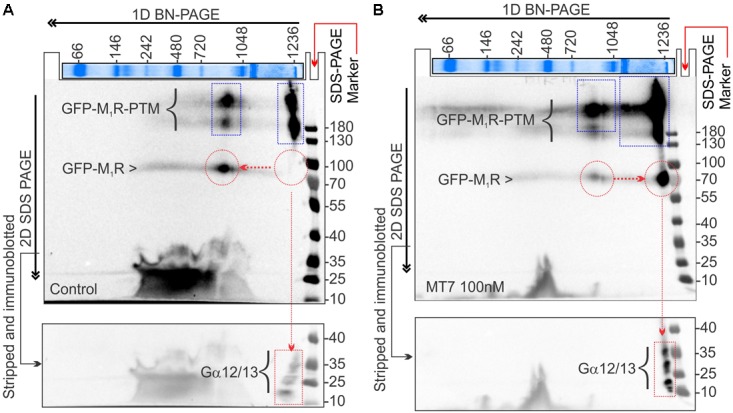
MT7 and pirenzepine elevated sequestration of trimeric G proteins in a protein complex associated with M_1_R in SH-SY5Y cells. **(A,B)** BN-PAGE analysis showing recruitment and sequestration of G proteins to M_1_R following MT7 treatment. GFP-M_1_R transfected cells were treated with 100 nM MT7 and incubated for 1 h. Cell lysates were then separated on 1D BN PAGE followed by 2D SDS-PAGE, **(A)** Control, **(B)** 100 nM MT7 treatment. Top panel: Coomassie stained gel piece showing 1D BN-PAGE separation of native page protein molecular weight marker. The red horizontal and vertical arrows indicate the direction of the 1D BN-PAGE and 2D SDS-PAGE, respectively. The blue rectangle shows PTMs of GFP-M_1_R associated with 1000 kDa and >1200 kDa MPCs. The red circle indicates native form of the GFP-M_1_R associated with the MPCs. The red arrow connecting the red circles indicates shift of molecular weight in MPC due to recruitment of G proteins following drug treatment. The red rectangle in the bottom panel shows the co-migration of possible interacting G proteins with the MPCs.

Adult DRG cultures have very low cell yields, in the range of 250,000 per culture. To enable feasible pull down of protein complexes we used human neuroblastoma SH-SY5Y cultures that allow use of millions of cells. This cell line also exhibits a cholinergic phenotype with ACh secretion, expression of muscarinic receptors and AChE shedding (Yamada et al., 2011). We examined the recruitment of trimeric G proteins to M_1_R by Halo-pull down assay and BN-PAGE analysis (**Figures 8**, **9**). The Halo-pull down assay permitted a focus on the over-expressed M_1_R with no contamination from endogenous muscarinic receptors of mixed sub-type. The SH-SY5Y cells that overexpressed Halo-M_1_R were treated with drugs and Halo-tagged M_1_R was pulled down using Halo-linked resin. Subsequently, the pull down product was resolved in SDS-PAGE and immunoblotted with anti-M_1_R, anti-Gα12/13 and anti-Gγ/2/3/4/7 antibodies (**Figures 8B–D**). The Halo-M_1_R pull down fraction from the drug treated cells showed significantly elevated levels of Gγ/2/3/4/7 and Gα12/13 proteins when compared with untreated cells (**Figures 8E,F**).

The 2D BN-PAGE/SDS-PAGE analysis revealed existence of 2 protein complexes at ∼900 and ∼1200 kDa equivalent molecular weights in Halo-M_1_R over-expressing SH-SY5Y cells (**Figures 9A,B**). Each complex was associated with a native ∼100 kDa Halo-tagged M_1_R protein and >180 kDa fractions, the latter may be derived from different PTMs of the Halo-M_1_R. Treatment with 100 nM MT7 caused a major shift of the ∼900 kDa protein complex to the ∼1200 kDa protein complex within 1 h of treatment, suggestive of recruitment of putative interacting proteins (**Figure 9**, blue and red dotted areas). In contrast, untreated cells did not show any shift in the ∼900 kDa protein complex to the ∼1200 kDa protein complex indicating less or no recruitment of interacting proteins (**Figure 9A**). Further, when the same blots were immunoblotted with anti-Gα12/13 antibodies, the Gα12/13 proteins appeared as spots on a vertical line corresponding to the ∼1200 kDa protein complex in the drug treated cells which suggests possible co-migration and association with M_1_R (**Figures 9A,B**, bottom panel).

## Discussion

Our recent work has shown that sensory neurons derived from M_1_R null mice exhibit enhanced neurite outgrowth (Calcutt et al., 2017). We now demonstrate that over-expression of M_1_R inhibited neurite outgrowth, caused disruption of the tubulin cytoskeleton and blockade of mitochondrial trafficking in adult sensory neurons, all of which were rescued by exposure to selective or specific M_1_R antagonists. We then used overexpression of GFP-M_1_R to identify the molecular pathway components associated with specific M_1_R-mediated cellular phenotypes. Based on our data, we propose that ACh mediated signaling via M_1_R constrains neurite outgrowth via activation of Gα13 proteins, which in turn limits tubulin polymerization and mitochondrial trafficking within axons.

Overexpression of M_1_R in sensory neurons has biological consequences that likely arise from the presence of neuron-derived ACh in the culture environment. Cultured sensory neurons secrete endogenous ACh into the extracellular media to generate a local concentration in the range of approximately 16 μM (Calcutt et al., 2017). This far exceeds the ACh *K*_d_ of 25–35 nM measured in several regions of rat brain tissue (Kellar et al., 1985) or the *K*_d_ of 0.2–0.4 nM measured using rat brain neurons (Pavia et al., 1991; Bakker et al., 2015). In the presence of abundant extracellular ACh, overexpressed. M_1_R will trigger activation of Gα13 proteins that leads to the dissociation of tubulin microtubules, as seen in **Figure 4**. The chemogenetically modified M_1_R-DREADD mutant significantly reduced M_1_R induced growth retardation (**Figure 2**). Thus, ACh-driven basal activity of M_1_R is responsible for neurite outgrowth suppression under conditions of M_1_R over-expression. In addition, we propose in normal un-transfected neurons that basal endogenous M_1_R signaling tonically suppresses neurite outgrowth by restricting mitochondrial (and potentially vesicular) transport, thereby explaining the ability of antimuscarinic drugs to prevent/reverse this constraint.

Knockdown of Gα13 in M_1_R-overexpressed neurons significantly reversed the M_1_R-induced growth inhibitory effect and protected from tubulin destabilization in growth cones (**Figure 7**). Gα13 is more abundant than Gα12 in sensory neurons and M_1_R was linked to activation of Gα12/13 type G proteins that leads to activation of small GTP binding protein Rho through mobilization of RhoGEF (Luo et al., 2001; Siehler, 2009; Haga, 2013). Activation of Gα12/13 leads to stimulation of the Rho/Rho Kinase pathway via a subgroup of Rho guanine nucleotide exchange factors (Fukuhara et al., 2001). Involvement of Rho and ROCK (Rho-associated coiled coil forming protein kinase) in agonist-induced neurite retraction and cell rounding has been reported in N1E-115 neuroblastoma cells (Hirose et al., 1998). Dominant-negative p160-ROCK completely abolished this neurite retraction suggesting a clear link between RhoA-ROCK signaling and cytoskeleton disassembly (Hirose et al., 1998). Activation of neuronal cannabinoid receptors linked to Gα12/13 proteins triggered rapid and reversible contraction of actinomyosin cytoskeleton through a Rho-GTPase and ROCK (Roland et al., 2014). Further, the CRISPR/Cas9 based knockout of Gα12/13 proteins in the HEK293 cell line augmented the abundance of polymerized tubulin (**Supplementary Figure S1**). HEK293 cells have been utilized as a model system for neuronal synapse formation (Biederer and Scheiffele, 2007) as they express neuronal proteins and have neuronal cell-lineage (Shaw et al., 2002; Stepanenko and Dmitrenko, 2015). Using a NanoBIT split luciferase based RhoA biosensor that detects Gq-induced RhoA activation showed that the RhoA signal is completely lost in the Gα12/13 KO cell (Mercier et al., manuscript in revision, personal communication).

**FIGURE 10:**
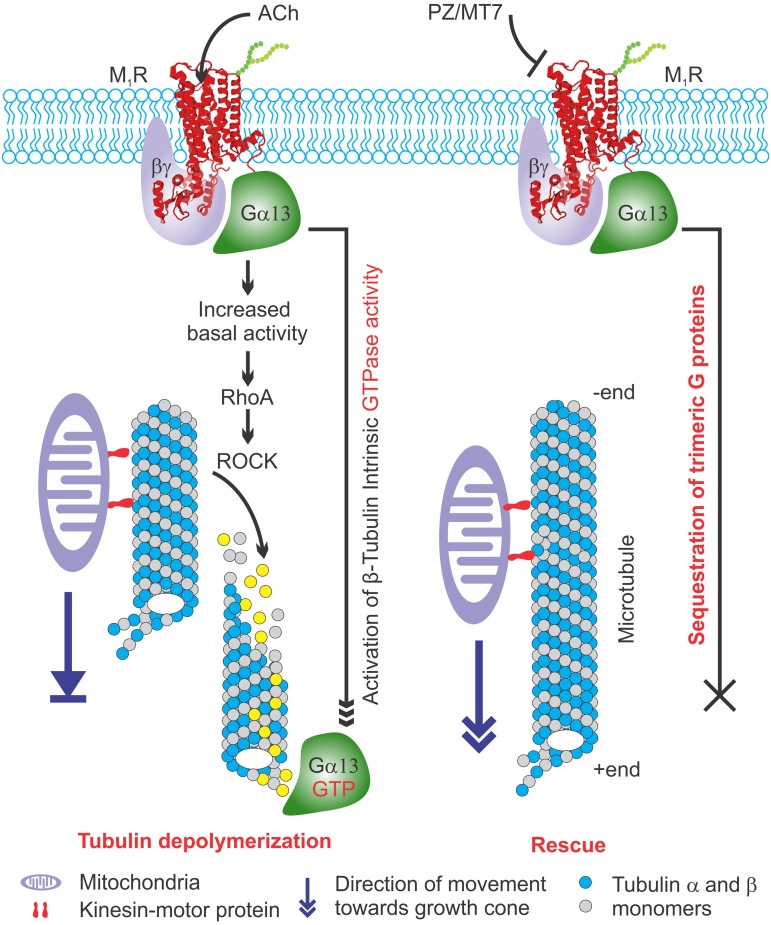
Model explaining the effect of M_1_R overexpression and antagonism on tubulin associated cytoskeleton and mitochondrial trafficking. **(Left)** M_1_R overexpressing neurons have increased basal activity in response to secreted acetylcholine signaling through overexpressed receptor. This is turn causes recruitment of trimeric G proteins that destabilize tubulin polymers by increasing intrinsic GTPase activity of tubulin. The lack of tubulin cytoskeleton in M_1_R overexpressing neuron leads to decreased mitochondrial trafficking and stagnation of mitochondria in the neurites that impairs outgrowth. **(Right)** M_1_R overexpression in the presence of antagonists MT7 and pirenzepine (PZ) stabilizes tubulin polymerization. The antagonists bind to the M_1_R and may stabilize a specific structural ensemble, which in turn recruit trimeric G proteins. However, the antagonist mediated M_1_R structural ensemble may sequester the bound G proteins and makes them unavailable for exerting their effect on tubulin polymerization which in turn stabilizes microtubule cytoskeleton and promotes mitochondrial trafficking and neurite outgrowth.

Activated G proteins regulate tubulin polymerization (Schappi et al., 2014) and tubulin binds directly to Gα or Gβγ subunits (Wang et al., 1990; Roychowdhury et al., 1999; Sarma et al., 2003). Activated GTP bound Gα promotes microtubule instability by increasing the intrinsic hydrolysis of GTP-tubulin to the less polymer stable GDP-tubulin (Roychowdhury et al., 1999, 2006). We have modelled this in **Figure 10**. Overexpression of Gα_q_ in a rat pituitary cell line showed a 50% decrease in the ratio of soluble to polymerized tubulin (Ravindra et al., 1996). There is considerable cell type and isoform specificity in Gα mediated tubulin cytoskeleton dynamics (Sarma et al., 2015). We found that Gα12/13 and Gγ(2,3,4,7) were sequestered upon M_1_R antagonist binding (**Figures 8**, **9**) and it is plausible that these factors also regulate tubulin polymerization in sensory neurons. In addition, some guanine nucleotide exchange factors (GEFs) for Rho GTPases, namely p115 RhoGEF (Kozasa et al., 1998), PDZ-RhoGEFs (Fukuhara et al., 1999), and LARG (Suzuki et al., 2003) can act as direct couplers of Gα12/13 proteins to small GTPases such as RhoA, Rac1, and CDC42, all of which are known to influence microtubule dynamics (Hall and Lalli, 2010). The Gα12/13-RhoGEF-RhoA pathway of GPCR has been implicated in many diseases (Siehler, 2009). Interestingly, while Gα promotes tubulin disassembly by increasing the tubulin GTPase activity, Gβγ subunits preferentially associate with GDP-bound tubulin to promote polymerization and stability of the microtubule (Roychowdhury and Rasenick, 1997; Popova and Rasenick, 2003; Roychowdhury et al., 2006). Our data from non-crosslinked halo-pull down (**Figure 8**) and BN-PAGE analyses (**Figure 9**) show augmented association and occupancy of trimeric G-proteins (Gα12/13) to M_1_R during MT7 or PZ treatment, which indicates elevated sequestration of these proteins on M_1_R. We propose that muscarinic antagonist-induced sequestration of trimeric G-proteins restricts their dissociation from the overexpressed M_1_R associated protein complex and thereby limits their detrimental effect on tubulin polymerization (see **Figure 10**). Further, we posit that the same M_1_R suppression pathway is occurring in normal un-transfected neurons to limit cytoskeleton formation in axons and is counteracted by these drugs. However, this does not exclude the possibility that other pathways may be involved. For example, Ca^2+^ signaling/homeostasis are known to be responsible for the maintenance of cytoskeletal integrity (Tsai et al., 2015). Cholinergic activation of M_1_R coupled with the Gq/11 protein generates cytosolic calcium transients via phospholipase-C signaling pathway (Langmead et al., 2008). High Ca^2+^ may act to increase the intrinsic GTP hydrolysis of tubulin and directly destabilize growing microtubule ends without changing the effective concentration of tubulin (O’Brien et al., 1997). Therefore, it is possible that excessive cholinergic signaling may imbalance intracellular Ca^2+^ homeostasis and promotes tubulin destabilization. Antagonist mediated sequestration of G-proteins may limit this response and protect Ca^2+^ induced tubulin destabilization. However, further experimentation is required to prove this hypothesis. Insertion and site-directed mutagenesis based studies have revealed potential G-protein interaction sites in the i2 and i3 loops in mAChRs (Blin et al., 1995; Liu et al., 1996; Hu et al., 2010). Overexpression of mutated M_1_R for disruptive Gα13 binding and subsequent reversal of cholinergic tubulin destabilization effect would provide another means of testing. The exact binding site for Gα13 protein in M_1_R needs to be evaluated.

Overexpression of M_1_R restricted the number of active mitochondria in neurites, presumably through diminished trafficking as a direct consequence of overexpressed M_1_R-induced disruption of the cytoskeleton. The M_1_R antagonists, pirenzepine and MT7, were able to protect mitochondrial transport. Pirenzepine and MT7 also enhance mitochondrial oxygen consumption rate and respiratory complex activities through activation of the AMP-activated protein kinase (AMPK)/peroxisome proliferator-activated receptor γ coactivator-1α signaling axis (Calcutt et al., 2017). Mitochondrial function was also enhanced in sensory neuron cultures derived from M_1_R null mice. Thus, ACh signaling through M_1_R can negatively regulate mitochondrial phenotype at multiple levels in the neuron that include trafficking and positioning as well as fine regulation of activity of the respiratory complexes. Optimal regulation of mitochondrial function is critical in distal regions of sensory neurons which, in human peripheral nerve, can be up to a meter from the cell body (Schwarz, 2013). The architecture of sensory neurons poses an extreme cellular environment for mitochondrial distribution and a need to supply energy to the distal endings where energy demand is high (Bernstein and Bamburg, 2003; Chowdhury et al., 2013). Any defect in mitochondrial function is likely to have a profound influence on the axon. Recent *in vivo* studies of mitochondrial transport along the saphenous nerve of adult mice revealed elevated anterograde transport of this organelle in axons undergoing high rates of depolarization and impulse conduction (Sajic et al., 2013). Indeed, loss of function of mitochondrial proteins such as *bcl-w* or mitofusin-2 results in a length-dependent dying-back sensory neuropathy (Baloh et al., 2007; Misko et al., 2010; Courchesne et al., 2011). Genetic ablation of mitochondrial transport also leads to axonal growth failure following axotomy in mice (Zhou et al., 2016). In axons, approximately 30–40% of total mitochondria are constantly engaged in saltatory motion (Lovas and Wang, 2013). It is plausible that the microtubule disruption induced by overexpression of M_1_R eliminated the basic framework for motor proteins to carry their mitochondrial cargo and potentially other vesicular cargos. As a result, the physical abundance of active mitochondria was diminished in the distal neuritis, which would be expected to deprive the neuronal growth cone of an essential supply of ATP, resulting in suppressed actin treadmilling and neurite outgrowth. This hypothesis, summarized in **Figure 10**, is further supported by reports that several microtubule-targeted chemotherapeutic agents, such as colchicine and vincristine, are known to induce a sensory neuropathy in which the distal aspect of the sensory axon gradually degenerates (Bennett et al., 2014).

Our pre-clinical studies have demonstrated that selective and specific M_1_R antagonists promote neurite outgrowth in adult sensory neurons *in vitro* (Calcutt et al., 2017). MT7 and pirenzepine were also able to prevent or reverse the distal degenerative neuropathy characteristic of diabetes, chemotherapy-induced and HIV-induced peripheral neuropathy. In the present study, we have reinforced the finding that M_1_R antagonists are neuronal growth promoters using an unbiased automated high throughput neurite outgrowth measurement technique in a large cohort of sensory neurons. In addition, we have highlighted a molecular mechanism that indicates that treatment with these drugs stabilizes microtubules by sequestering Gα13 proteins and promotes microtubule-based axonal transport of mitochondria, which in turn augments neurite outgrowth. Peripheral neuropathy is a major cause of human morbidity with huge associated health care costs (Gordois et al., 2003; McInnes, 2012). One particularly encouraging implication of our identification of the endogenous M_1_R-mediated suppression of axonal outgrowth in sensory neurons is that antimuscarinic drugs that can prevent/reverse this process have already been widely used as approved drugs for other indications (Siatkowski et al., 2008). Since small fiber axonal degeneration is an early feature of many peripheral neuropathies, the novel growth-regulating pathway we have identified could be mobilized to prevent or reverse distal neurodegeneration. Our studies support a previously unrecognized therapeutic potential for M_1_R antagonists in the treatment of peripheral neuropathies and unravels a novel pathway of cholinergic signaling mediated via control of microtubule dynamics through Gα13 signaling.

## Author Contributions

MS conceptualized, designed and performed all the experiments, analyzed all data, prepared all figures, and wrote the manuscript. PF and NC obtained funding to support the work, aided in the design of experiments, and contributed to writing and editing of the manuscript.

## Conflict of Interest Statement

NC and PF declare that they are scientific founders of, have an equity interest in, WinSanTor Inc., a company that has licensed IP from the University of Manitoba and University of California San Diego and may potentially benefit from the research contained herein. The terms of this arrangement have been reviewed and approved by the University of California, San Diego in accordance with its conflict of interest policies. PF also serves on the Board of Directors of WinSanTor Inc. The remaining author declares that the research was conducted in the absence of any commercial or financial relationships that could be construed as a potential conflict of interest.

## Footnotes

1 www.proteinatlas.org

## Supplementary Material

FIGURE S1 Polymerized α-tubulin is augmented in Gα12/13 null HEK293 cells. **(A)** Immunoblots showing expression of Gα12, Gα13, and porin (VDAC1) in wild type and Gα12/13 null HEK293 cell. **(B)** Immunoblots showing the relative amount of polymerized tubulin in wild type and Gα12/13 null HEK293 cells. **(C)** In BN-PAGE based assay, polymerized tubulin was stabilized in microtubule stabilization buffer and resolved in native gradient gel. The polymerized tubulin microtubules are trapped in the native page at the top (∼1200 kDa) whereas the dimers and monomers migrated to lower molecular weight regions. The first dimension gel was denatured and the proteins were further separated in 2nd dimension SDS-PAGE and immunoblotted. The spots at ∼146 and ∼66 kDa represent α-tubulin dimer and monomers. The red dotted circle represents the polymerized tubulin. Green and blue arrows indicate direction of protein movement. **(D)** Scatter plot showing polymerized tubulin in wild type and Gα12/13 null HEK293 cell as revealed by polymerized tubulin and BN-Page based assays. In the BN-PAGE assay the polymerized tubulin was quantified based on the relative intensity of the monomeric tubulin. *N* = 3/4 independent experiments. Data represented as mean ± SEM, *p*-value by unpaired *t*-test. **(E)** Immunofluorescent images showing actin (phalloidin) and (α-tubulin) in HEK293 (Wild type: WT) and GNAS12/13 knockout (ΔGNAS12/13) cells. Scale bar: 20 μm.

FIGURE S2 Actin and tubulin cytoskeleton in wild type and G12/13 KO cells. **(A,B)** Confocal immunofluorescent images showing F-actin (phalloidin stained, top panel, **A**) and tubulin (α-tubulin immunolabelled, bottom panel, **B**) cytoskeleton in GFP-M_1_R expressing wild type and G12/13 KO cells. Blue arrow indicates focal adhesions, yellow arrow indicates extended cytoplasmic processes enriched in tubulin. Scale bar: 20 μm.

FIGURE S3 Effect of carbachol on tubulin cytoskeleton in M_1_R expressed wild type and G12/13 KO cells. Confocal immunofluorescence images showing the tubulin cytoskeleton. Yellow arrow indicates crest of the cell with dense packing of tubulin. White and blue arrows indicate elongated cytoplasmic processes enriched in tubulin. Blue arrow indicates localization of GFP-M_1_R in extended cytoplasmic processes. *Z* represents a particular optical slice in *Z*-stacked image series. Scale bar: 20 μm.
