# Supplementary figures and images for: Muscarinic Acetylcholine Type 1 Receptor Activity Constrains Neurite Outgrowth by Inhibiting Microtubule Polymerization and Mitochondrial Trafficking in Adult Sensory Neurons
Source: Front Neurosci. 2018 Jun 26;12:402. doi: 10.3389/fnins.2018.00402 (PMC6029366; doi:10.3389/fnins.2018.00402)

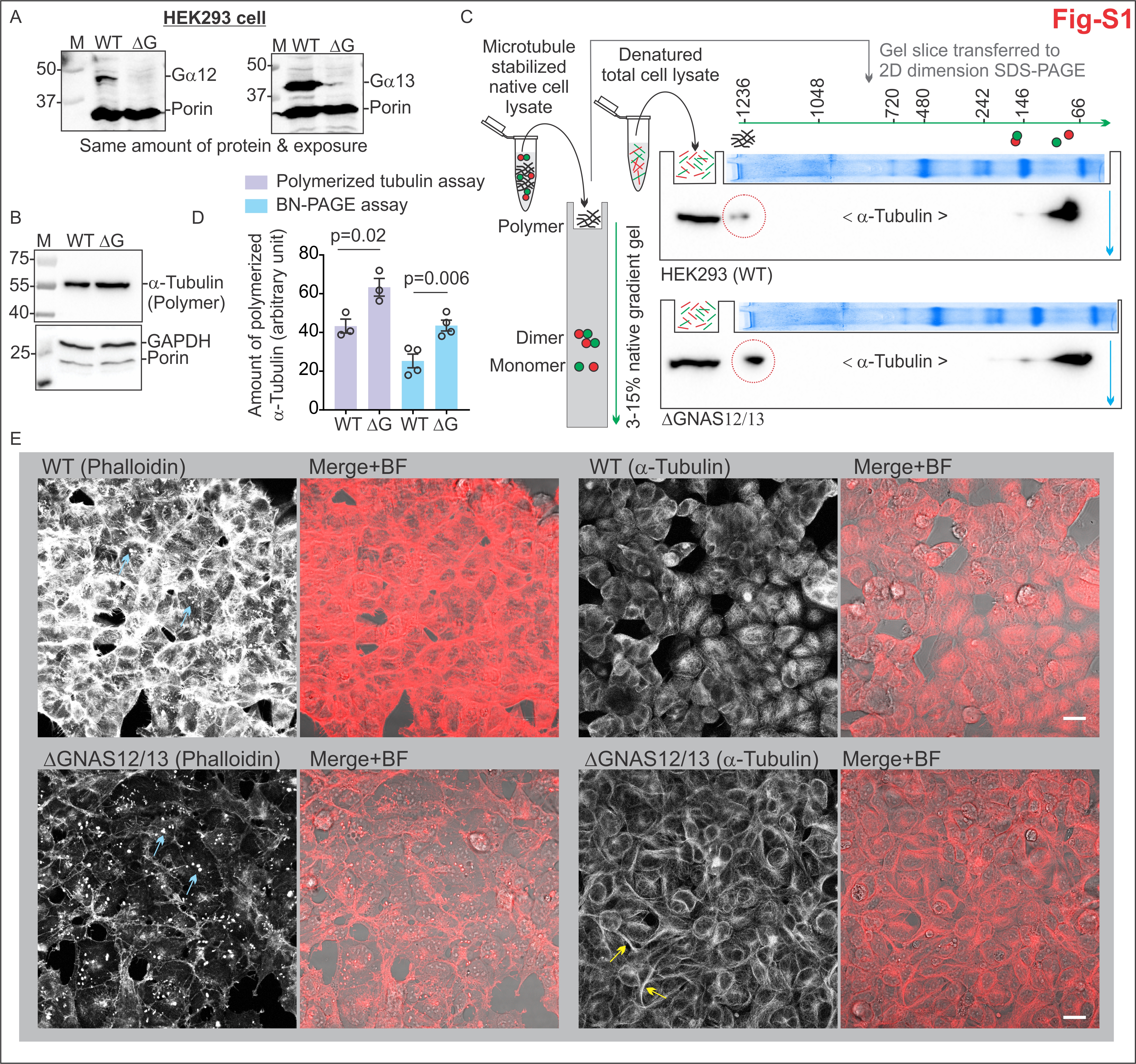

Supplement: FIGURE S1 — Polymerized α-tubulin is augmented in Gα12/13 null HEK293 cells. (A) Immunoblots showing expression of Gα12, Gα13, and porin (VDAC1) in wild type and Gα12/13 null HEK293 cell. (B) Immunoblots showing the relative amount of polymerized tubulin in wild type and Gα12/13 null HEK293 cells. (C) In BN-PAGE based assay, polymerized tubulin was stabilized in microtubule stabilization buffer and resolved in native gradient gel. The polymerized tubulin microtubules are trapped in the native page at the top (∼1200 kDa) whereas the dimers and monomers migrated to lower molecular weight regions. The first dimension gel was denatured and the proteins were further separated in 2nd dimension SDS-PAGE and immunoblotted. The spots at ∼146 and ∼66 kDa represent α-tubulin dimer and monomers. The red dotted circle represents the polymerized tubulin. Green and blue arrows indicate direction of protein movement. (D) Scatter plot showing polymerized tubulin in wild type and Gα12/13 null HEK293 cell as revealed by polymerized tubulin and BN-Page based assays. In the BN-PAGE assay the polymerized tubulin was quantified based on the relative intensity of the monomeric tubulin. N = 3/4 independent experiments. Data represented as mean ± SEM, p-value by unpaired t-test. (E) Immunofluorescent images showing actin (phalloidin) and (α-tubulin) in HEK293 (Wild type: WT) and GNAS12/13 knockout (ΔGNAS12/13) cells. Scale bar: 20 μm. [file Image_1.JPEG]

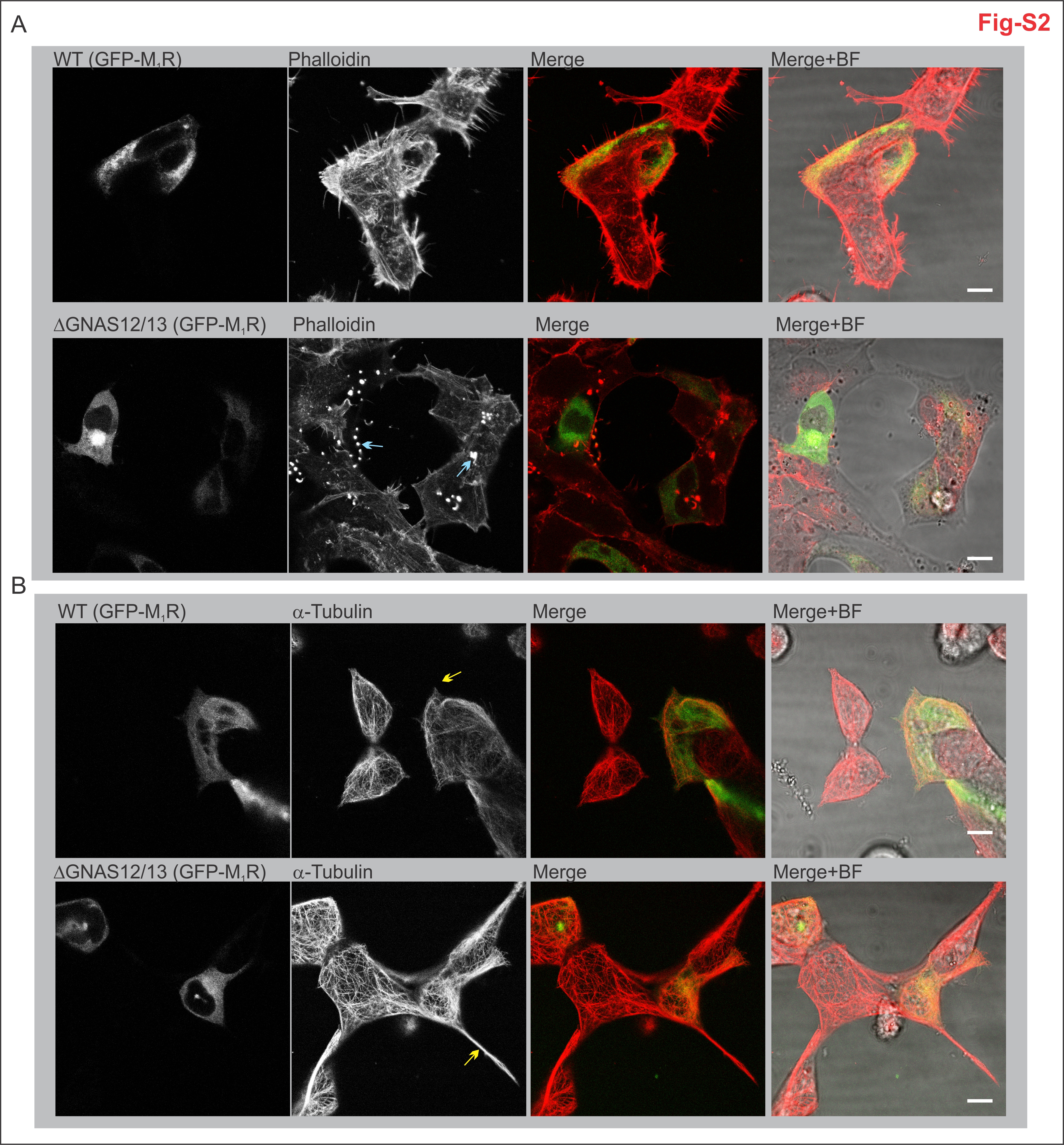

Supplement: FIGURE S2 — Actin and tubulin cytoskeleton in wild type and G12/13 KO cells. (A,B) Confocal immunofluorescent images showing F-actin (phalloidin stained, top panel, A) and tubulin (α-tubulin immunolabelled, bottom panel, B) cytoskeleton in GFP-M1R expressing wild type and G12/13 KO cells. Blue arrow indicates focal adhesions, yellow arrow indicates extended cytoplasmic processes enriched in tubulin. Scale bar: 20 μm. [file Image_2.JPEG]

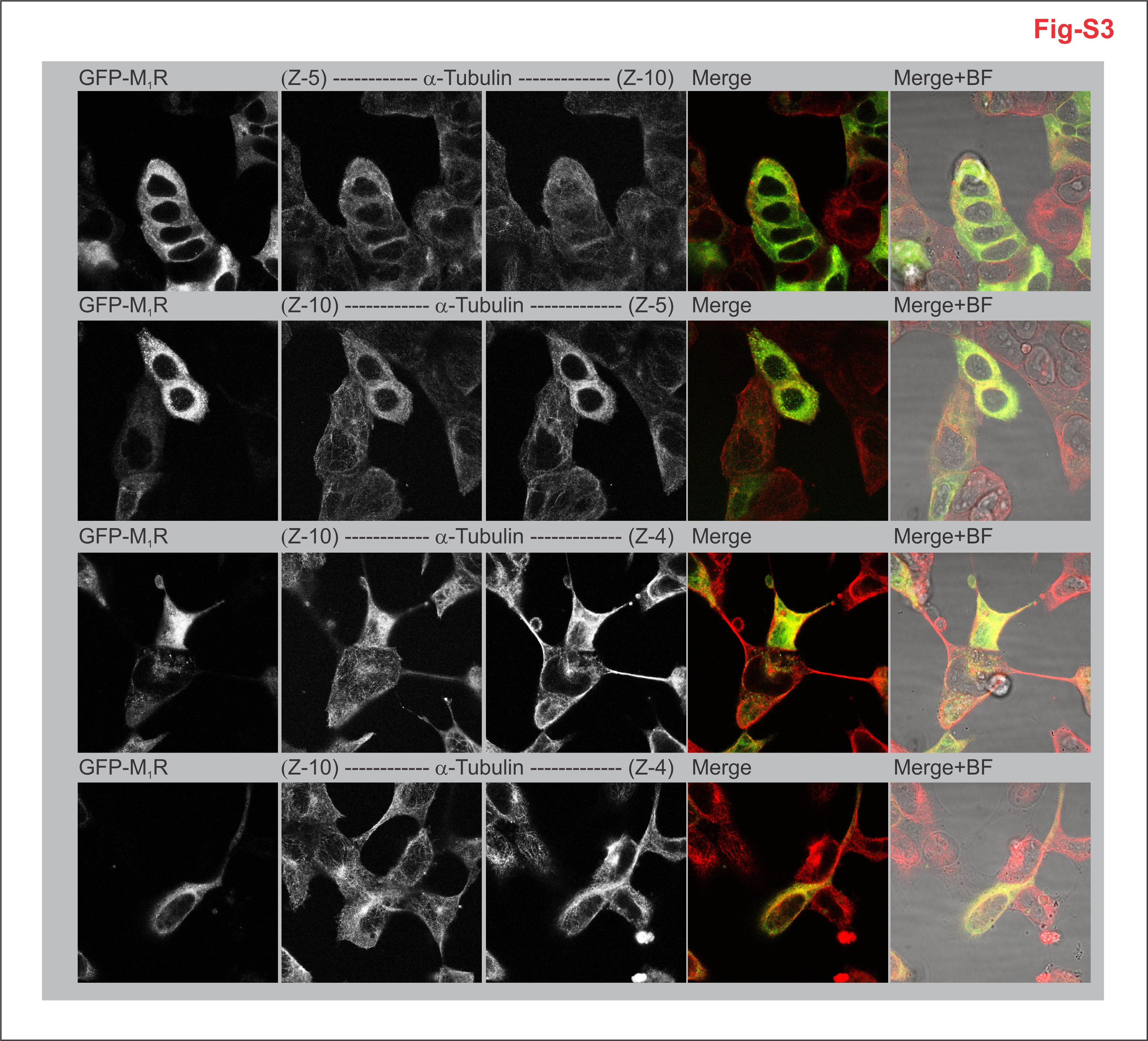

Supplement: FIGURE S3 — Effect of carbachol on tubulin cytoskeleton in M1R expressed wild type and G12/13 KO cells. Confocal immunofluorescence images showing the tubulin cytoskeleton. Yellow arrow indicates crest of the cell with dense packing of tubulin. White and blue arrows indicate elongated cytoplasmic processes enriched in tubulin. Blue arrow indicates localization of GFP-M1R in extended cytoplasmic processes. Z represents a particular optical slice in Z-stacked image series. Scale bar: 20 μm. [file Image_3.JPEG]
